# Protective Effect Conferred by Isometric Preconditioning Against Slow- and Fast-Velocity Eccentric Exercise-Induced Muscle Damage

**DOI:** 10.3389/fphys.2019.01203

**Published:** 2019-10-01

**Authors:** Renan Vieira Barreto, Leonardo Coelho Rabello de Lima, Camila Coelho Greco, Benedito Sérgio Denadai

**Affiliations:** ^1^Human Performance Laboratory, Department of Physical Education, São Paulo State University, Rio Claro, Brazil; ^2^Faculty of Biological and Health Sciences, University Centre Herminio Ometto, Araras, Brazil; ^3^School of Physical Education, Salesian University Centre of São Paulo, Campinas, Brazil

**Keywords:** muscle damage, eccentric exercise, repeated bout effect, strength loss, delayed-onset muscle soreness, index of protection

## Abstract

We investigated if the same isometric preconditioning protocol (IPP) attenuates the magnitude of muscle damage induced by different maximal eccentric exercise protocols in the elbow flexors. Sixty-four untrained men were assigned to either two experimental or two control groups. Participants in the experimental groups performed an IPP prior to either slow (60°·s^−1^ – ISO + ECC-S) or fast (180°·s^−1^ – ISO + ECC-F) maximal eccentric contractions (MaxECC). Subjects in the control groups performed slow (ECC-S) or fast (ECC-F) MaxECC without IPP. Maximal isokinetic concentric torque (MVC), muscle soreness (SOR), and muscle thickness (MT) were assessed before, immediately after, and 1–4 days following the MaxECC. Significant (*p* < 0.05) group vs. time interactions were found for MVC (*F* = 4,517), SOR (*F* = 6,318), and MT (*F* = 1,863). The ECC-S group presented faster (*p* < 0.05) recovery of MVC and MT and less (*p* < 0.05) SOR at 96 h post-MaxECC compared with ECC-F group. No significant differences in MVC and MT were found between ECC-S and ECC-F groups following MaxECC. The ISO + ECC-S group showed faster (*p* < 0.05) recovery of MVC and SOR compared to the ECC-S group. No significant differences were evident between ISO + ECC-S and ECC-S in any variable. The ISO + ECC-F group showed faster (*p* < 0.05) recovery of all assessed variables compared with the ECC-F group. MVC was greater (*p* < 0.05) at 48–72 h, and SOR was less (*p* < 0.05) at 48–96 h in the ISO + ECC-F compared to the ECC-F group. No significant differences were evident between ISO + ECC-S and ISO + ECC-F for any variable. These results show that the IPP accelerated recovery of MVC and SOR for the slow-eccentric exercise condition and attenuated strength loss and SOR in addition to faster recovery of all assessed variables for the fast-eccentric exercise condition. Therefore, the IPP can be used as a strategy to attenuate and accelerate recovery of muscle damage induced by different-velocity eccentric exercises, resulting in greater protection against muscle damage induced by faster velocity.

## Introduction

Unaccustomed exercise, especially those involving eccentric contractions, causes ultrastructural disruptions in skeletal muscle fibers termed as exercise-induced muscle damage (EIMD) and leads to adaptations that make the muscle less vulnerable to damage on a subsequent similar exercise (Clarkson and Hubal, 2002; Hyldahl and Hubal, 2013). This protective effect is referred to as the repeated bout effect (RBE), and it is evidenced by faster recovery of muscle function and blunted changes on EIMD markers (i.e., muscle swelling, delayed onset muscle soreness, and edema) following a repeated exercise bout (Hyldahl et al., 2017). Although EIMD is a natural phenomenon and it is attenuated through repeated exercises sessions, some of its symptoms may acutely affect performance and the well-being of different populations (Assumpção et al., 2013; Nelson, 2013; Peñailillo et al., 2015).

Recent studies have demonstrated that the RBE can be conferred even when the initial exercise bout does not induce significant EIMD (for review, see Nosaka and Aoki, 2011). For instance, Lavender and Nosaka (2008) showed that submaximal non-damaging eccentric exercise of the elbow flexors conferred a protective effect against a more intense eccentric damaging protocol. Indeed, it has been reported that maximal voluntary isometric contractions (MaxISO) induce a potent protective effect against EIMD caused by eccentric exercise performed a few days later with the same muscle group (Chen et al., 2012a,b, 2013) or the contralateral homologous muscle group (Chen et al., 2018). Interestingly, this protective effect manifests without the occurrence of previous histological damage, and its magnitude depends on the muscle length at which the MaxISO are performed (Chen et al., 2012b), total volume of MaxISO (Chen et al., 2012a), and the interval between the isometric preconditioning protocol (IPP) and the damaging bout (Chen et al., 2013).

Chen et al. (2012b) found greater protective effect when IPP was performed at a long muscle length (20° of elbow flexion) as compared with the IPP performed at a short muscle length (90° of elbow flexion). Moreover, this study showed that 30 MaxISO induced a protective effect that lasted as long as 3 weeks. However, the investigated IPP led to significant alterations on indirect markers of EIMD (Chen et al., 2012b). Accordingly, Chen et al. (2012a) showed that 2 or 10 MaxISO attenuated the magnitude of changes in EIMD markers induced by 30 MaxECC at an angular velocity of 90°·s^−1^, with greater protective effect conferred by 10 MaxISO. In another study, Chen et al. (2013) showed that the protective effect conferred by 2 MaxISO is not long lived, lasting up to 4 days with a peak of protection occurring 2 days after the IPP.

To our knowledge, all studies investigating IPP as a strategy to attenuate subsequent EIMD have been published by the same research group (Chen et al., 2012a,b, 2013), which tested different IPPs against similar damaging protocols. These studies suggest that performing a low volume of MaxISO at a long muscle length 2 days before an eccentric damaging exercise bout can be an interesting strategy to accelerate recovery and/or attenuate EIMD symptoms. Nevertheless, the magnitude of EIMD can be influenced by exercise characteristics such as intensity (Paschalis et al., 2005), number of contractions (Chen and Nosaka, 2006), angular velocity (Chapman et al., 2006, 2008) or joint angle (Nosaka and Sakamoto, 2001) during the eccentric contractions, which vary among the different types of exercise training regimens, competitions, and daily activities.

In exercise training, contraction type and contraction velocity are the two major variables that influence exercise specificity and, therefore, exercise prescription. Regarding the effect of these variables on EIMD magnitude, it is widely accepted that eccentric contractions have greater potential to induce EIMD than concentric and isometric contractions (Clarkson and Hubal, 2002). Concerning the effect of contraction velocity on EIMD, previous studies indicated that faster eccentric contractions induce greater damage than slower eccentric contractions (Chapman et al., 2006, 2008). For instance, Chapman et al. (2006) found different magnitudes of changes in EIMD markers when untrained subjects performed slow eccentric exercise (30 MaxECC at 30°·s^−1^) with one arm followed by fast eccentric exercise (210 MaxECC at 210°·s^−1^) with the other arm 14 days after it. The authors chose different angular velocities of eccentric exercise to simulate the velocity of a general resistance training setting (slow eccentric contractions), or a sports situation (fast eccentric contractions), and the volume of contractions was prescribed to equate the total time under tension (120 s) among protocols. Is their study, the fast-eccentric exercise protocol induced greater decrease in strength production capacity and led to greater muscle soreness and swelling – with slower recovery of these markers – compared to the slow eccentric exercise protocol (Chapman et al., 2006). However, when Chapman et al. (2008) tested two different MaxECC velocities (30 and 210°·s^−1^) with an equalized number of contractions (30 or 210 contractions), the difference in the magnitude of EIMD between the two velocities was smaller. Therefore, the manipulation of both eccentric contraction velocity and number of eccentric contractions has a substantial effect on the magnitude of changes in EIMD markers and is a reasonable approach to investigate different EIMD magnitudes.

Given that the magnitude of EIMD varies according to eccentric exercise characteristics, which are different among exercises modalities, it is important to elucidate if IPP is an efficient strategy to attenuate EIMD symptoms induced by different eccentric exercise protocols. In a recent mini review by our research group (Lima and Denadai, 2015), a hypothetical model was proposed to explain the relationship between the number of MaxISO and the magnitude and duration of the resulting protection conferred against EIMD. We argue that the stress applied to the neuromuscular system during the IPP (number of contractions and muscle length) modulates the magnitude of the protective effect. A recent study investigating the protective effect conferred by MaxISO on EIMD induced in lower limb muscles (which are less susceptible to EIMD) illustrates this relationship (Tseng et al., 2016). In this study, a larger number of contractions (60 MaxISO) was necessary to induce protection against EIMD in the knee extensors compared to the number of contractions used in other studies (2–30 MaxISO) that investigated the effect of the IPP in the elbow flexors (that are more susceptible to EIMD). Thus, it seems reasonable to assume that the protection conferred by the same stimulus (same IPP) would not be the same against different extents of EIMD. No previous study has investigated the protective effect conferred by IPP against different eccentric exercise protocols or different extents of EIMD.

Therefore, the purpose of this study was to investigate if the same IPP attenuates the magnitude of muscle damage induced by different MaxECC protocols in the elbow flexors. We hypothesized that MaxISO would attenuate EIMD in both conditions, and the magnitude of the protective effect against mild EIMD, induced by slow-velocity MaxECC, would be greater than the magnitude of protection against greater EIMD, induced by fast-velocity MaxECC.

## Materials and Methods

### Subjects and Study Design

Sixty-four untrained young men who had not been engaged in any type of regular resistance training program in the previous 6 months participated in the present study. Their mean ± SD age, height, and body mass were 21.7 ± 3.1 years, 174 ± 5 cm, and 74.7 ± 13.6 kg, respectively. None of the subjects had any previous bone, joint or muscle injuries on the upper limbs. They did not consume any nutritional supplements or ergogenic aids prior to or during the experiment. All participants provided written informed consent to participate in the present study, which had been previously approved by the Institutional Ethics Committee. The present study was conducted in conformity with the policy statement regarding the use of human subjects by the Declaration of Helsinki.

Participants were assigned to one of two experimental or two control groups (*n* = 16 per group) by matching their baseline maximal voluntary isokinetic concentric peak torque. Subjects in the experimental groups performed the same IPP, which consisted of 10 MaxISO of the elbow flexors at a long muscle length (20° of elbow flexion, 0° = fully extension), 2 days prior to either slow- (60°·s^−1^ – ISO + ECC-S) or fast-velocity (180°·s^−1^ – ISO + ECC-F) maximal isokinetic eccentric contractions of the elbow flexors. Subjects in the control groups performed either slow- (ECC-S) or fast-velocity (ECC-F) eccentric contractions at the same angular velocities described above without any preconditioning protocol. All subjects used their dominant arm for all protocols and measurements. No significant differences in age, height, body mass, or maximal voluntary isokinetic concentric peak torque were evident among groups before the eccentric exercise.

The sample size was estimated using data collected in a pilot study examining the effects of 10 MaxISO of the elbow flexors at 20° of elbow flexion on changes in maximal voluntary concentric torque following 30 MaxECC at an angular velocity of 60°·s^−1^ performed 2 days later. It was estimated that a 10% difference would exist between groups for maximal voluntary concentric torque recovery at 96 h after MaxECC. Based on an effect size of 1.0, *α* level of 0.05, and a power (1 − β) of 0.80 (Cohen, 1988), it was estimated that a minimum of 14 subjects per group was necessary. We recruited two extra participants for each group considering the chance for dropouts during the experiment, which did not occur.

### Experimental Protocol

All assessments and interventions were performed at the same time of the day across the experimental period. Participants were familiarized with the testing procedures 3–7 days before the first exercise session (either isometric contractions or eccentric exercise). In the familiarization session, height, body mass, and maximal voluntary concentric peak torque of elbow flexors were assessed. The investigator showed the subjects how the isometric and eccentric contractions should be performed but no actual exercises were performed by the participants to avoid any degree of protection conferred by the familiarization session.

The dependent variables assessed in this study were maximal voluntary concentric peak torque (MVC), muscle soreness (SOR) assessed by visual analogue scale (VAS), and muscle thickness (MT) assessed by transverse B-mode ultrasound images. All these measurements were taken from the exercised arm. MVC and MT were taken immediately before, immediately after, and 24, 48, 72, and 96 h after the maximal eccentric exercise. SOR was measured at all time points shown above except immediately after the maximal eccentric exercise.

The test-retest reliability of the measures was established with a pilot study (*n* = 10) realized before the data collection of this study using the data taken 3–7 days and immediately before the first experimental session. Intraclass correlation coefficient (ICC) and coefficient of variation (shown in parentheses) for MVC and MT were 0.90 (6.6%) and 0.83 (6.7%), respectively.

### Isometric Preconditioning Protocol

Subjects in the experimental groups (ISO + ECC-S and ISO + ECC-F) performed 10 MaxISO with their elbow joints flexed at 20° (full extension = 0°) 2 days prior to maximal slow- or fast-velocity eccentric exercise, respectively. Each isometric contraction was sustained for 3 s and repeated every 45 s. They were seated on the isokinetic dynamometer (Biodex System 3 Pro, Biodex Medical Systems, Inc., Shirley, New York, USA) and had their hips and trunk fixed by straps, to guarantee the stability of the elbow joint and avoid compensation in the production of torque by other muscle groups. Participants’ upper arms were placed over an attachment that kept their shoulder joints flexed at 90° with 0° of abduction. Participants received strong verbal encouragement to apply as much torque as possible to the shaft during all contractions.

### Eccentric Exercises

Participants of the four groups underwent a maximal eccentric exercise session in the week following the familiarization session, respecting a 3–7-day interval between familiarization and eccentric exercise. The eccentric exercise protocol was performed on the isokinetic dynamometer, and the participants’ positioning was the same as described for the IPP.

The ECC-S and ISO + ECC-S groups performed a maximal eccentric exercise protocol consisting of 30 MaxECC, at an angular velocity of 60°·s^−1^ (i.e., slow velocity), with the elbow flexor muscles, whilst the ECC-F and ISO + ECC-F groups performed a maximal eccentric exercise protocol consisting of 90 MaxECC at an angular velocity of 180°·s^−1^ (fast-velocity), also with the elbow flexors. Eccentric contractions were divided into sets of 15 repetitions separated by 2-min recovery intervals. An interval of about 10 s between each eccentric contraction within the sets was also respected for the dynamometer lever arm to return to the initial position (at an angular velocity of 9°·s^−1^), with the aid of the examiner. A range of motion of 90° was adopted for both eccentric exercise protocols, starting from a semi-flexed position (90°) to the maximum extension (0°) of the elbow (Chen et al., 2013).

By choosing two different eccentric contraction velocities (60 or 180°·s^−1^), it was intended to induce different magnitudes of stress, and therefore EIMD, to participants’ elbow flexors (Chapman et al., 2006, 2008). However, previous studies suggest that the number of eccentric contractions has a stronger effect on changes in EIMD markers than velocity of contraction. Thus, we chose to equalize the time under tension of both eccentric exercise protocols (45 s) (Chapman et al., 2006). As a consequence, the fast-eccentric exercise protocol of the present study has a total number of contractions three times greater than the slow-eccentric exercise protocol, which was also intended to induce different magnitudes of EIMD.

### Dependent Variables

#### Maximal Voluntary Isokinetic Concentric Torque

Since the present study investigated the protective effects of isometric contractions on markers of EIMD, MaxISO were avoided whilst assessing strength production capacity. Hence, maximal voluntary isokinetic concentric torque was measured at the isokinetic dynamometer with the same participant positioning as described for the IPP at an angular velocity of 60°·s^−1^ and a range of motion of 120° of the elbow joint (0–120°) for three consecutive contractions. Verbal encouragement was provided during the tests. The peak torque of the three contractions was used for further analysis.

#### Muscle Soreness

SOR of the elbow flexors was assessed using a visual analogue scale (VAS) that had a 100 mm continuous line with “not sore” on one side (0 mm) and “very, very sore” on the other side (100 mm). The investigator asked the participants to rate their perceived soreness on the VAS whilst the muscles were palped and stretched by the volunteer himself. The reported values were measured with a ruler and registered for further analysis.

#### Muscle Thickness

MT was assessed by transverse B-mode ultrasound images taken using a portable ultrasound system (ProSound 2, ALOKA, Japan) with a 9.0 MHz linear probe and saved in a computer. Measures were taken at the mid portion of the upper arm, at half-distance between the acromion process of the clavicle to the lateral epicondyle of the humerus. The probe was positioned perpendicularly to the limb and coated with a generous amount of water-soluble transmission gel to provide acoustic contact between the skin and the transducer. All images were collected and analyzed with caution by the same investigator, avoiding compression of the dermal surface. Three measurements were performed. MT was determined using a computer with ImageJ 1.42q software (National Institutes of Health, Bethesda, Maryland). The mean of the three measurements of MT was considered for analysis.

### Index of Protection

To compare the protective effect conferred by IPP against EIMD by maximal eccentric exercises, indexes of protection (IP) were calculated for all variables during their peaks of manifestation for each group (Hyldahl et al., 2017). The IP was calculated from the following equation:

$$IP=\frac{\left(\Delta \%\mathrm{CON}-\Delta \%ISO\right)}{\Delta \%\mathrm{CON}}\times 100$$

where Δ% CON is the percentage change of the variable during its peak of manifestation in relation to the pre-exercise moment for the control groups (ECC-S or ECC-F), and Δ%ISO is the percentage change of the variable during its peak of manifestation in relation to the pre-exercise moment for the experimental groups (ISO + ECC-S or ISO + ECC-F). The IP was calculated for both velocities of eccentric exercise.

### Statistical Analyses

Data were analyzed using a statistical software package (SPSS Version 20.0; IBM, New York, NY). The normality of the data was confirmed by the Shapiro-Wilk test. Data homogeneity and sphericity were tested and confirmed by the Levene and Mauchly tests, respectively. Data are expressed as mean ± standard deviation, and the variance of the samples was investigated using two-way repeated-measures ANOVAs: time (6) vs. group (4). When a significant group vs. time interaction was identified, pairwise analyses were performed using one-way ANOVA for matched (time) and independent samples (group) followed by Bonferroni’s *post hoc*. The level of significance was set at *p* < 0.05 for all tests.

## Results

### Baseline Measurements

No significant differences between groups were found for baseline values of any of the assessed anthropometric and physiological characteristics (age, height, and body mass) (Table 1). The same occurred for the baseline values of all EIMD markers.

**Table 1:** Physiological and anthropometric characteristics.

	ECC-S	ISO + ECC-S	ECC-F	ISO + ECC-F
Age (year)	20.9 ± 2.8	22.2 ± 3.0	21.6 ± 2.3	22.0 ± 4.0
Height (cm)	174.3 ± 6.5	175.5 ± 4.2	174.2 ± 5.2	173.0 ± 4.1
Body mass (kg)	80.5 ± 16.6	74.9 ± 11.7	73.1 ± 13.5	70.4 ± 11.2
MVC (N·m)	50.7 ± 13.4	49.1 ± 11.2	50.9 ± 10.0	49.8 ± 13.8
SOR (mm)	0.0	0.0	0.0	0.0
MT (mm)	33.7 ± 5.5	33.9 ± 4.7	33.2 ± 7.0	33.4 ± 4.3

*MVC, maximal voluntary concentric peak torque; SOR, muscle soreness; MT, muscle thickness; ECC-S, control group of slow-eccentric exercise condition; ISO + ECC-S, experimental group of slow-eccentric exercise condition; ECC-F, control group of fast-eccentric exercise condition; ISO + ECC-F, experimental group of fast-eccentric exercise condition. No significant differences between groups were found for baseline measures (*p* > 0.05)*.

### Isometric Preconditioning

**Figure 1:**
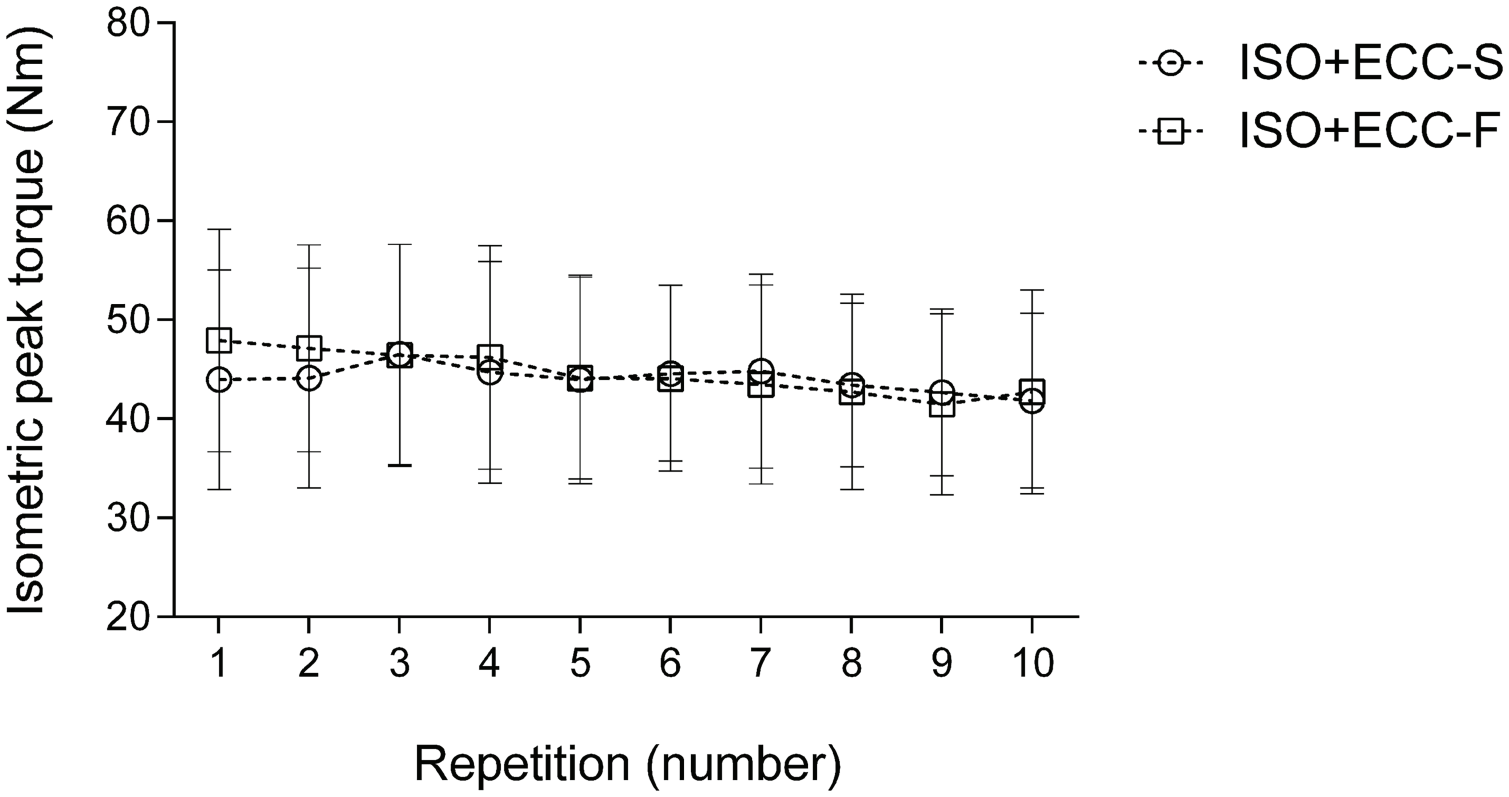
Changes (mean ± SD) in isometric peak torque over the repetitions during isometric preconditioning protocol for the experimental groups (ISO + ECC-S and ISO + ECC-F). No significant differences over repetitions were found between groups (*p* > 0.05).

Average peak torque of the elbow flexors during the IPP was similar between experimental groups (ISO + ECC-S: 43.8 ± 9.0 N·m, ISO + ECC-F: 44.5 ± 10.2 N·m) (*p* = 0.86). Figure 1 shows isometric peak torque produced during each MaxISO of the IPP. No significant differences over repetitions were found between groups (*p* > 0.05).

### Eccentric Exercises

**Figure 2:**
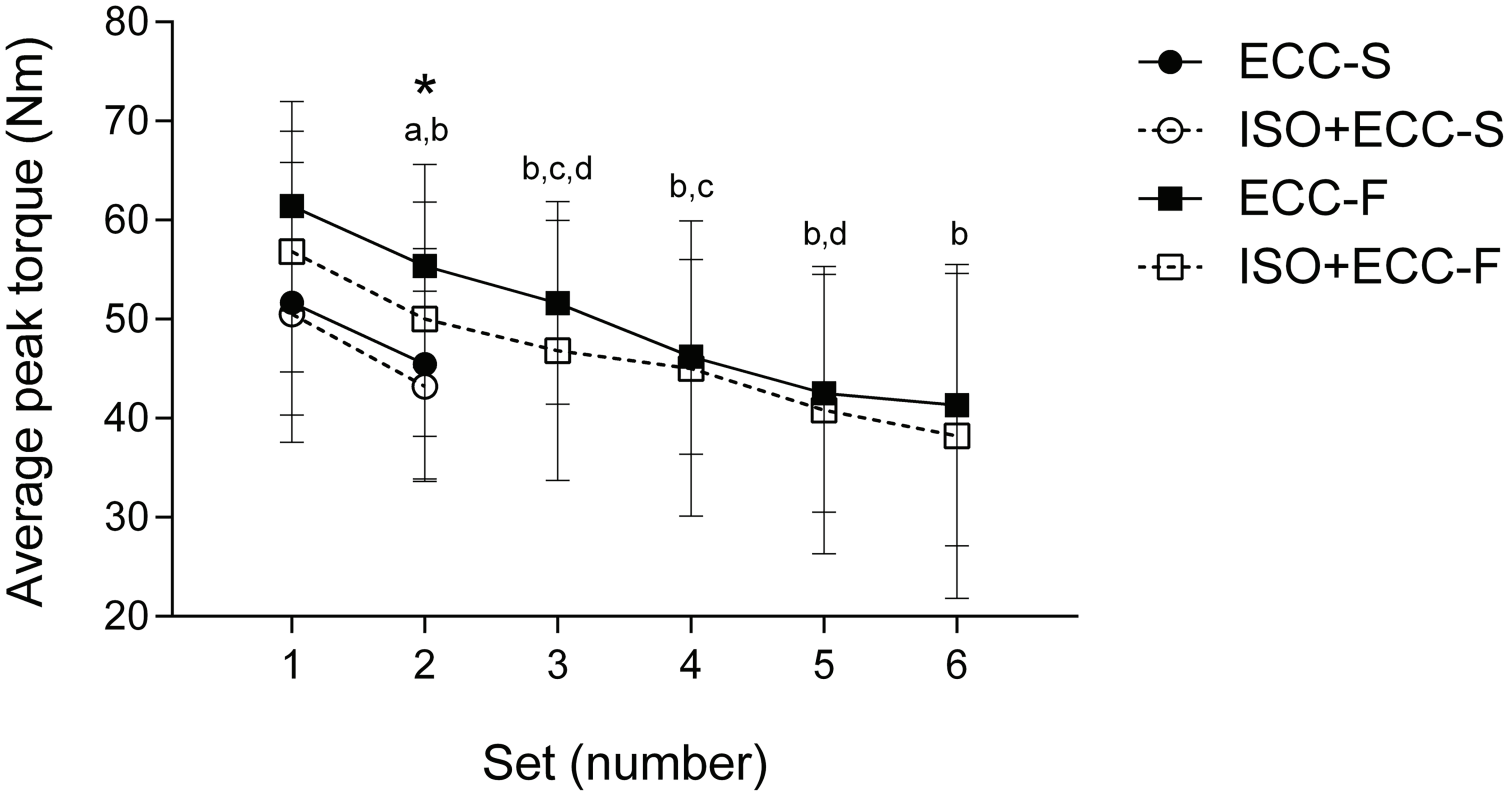
Changes (mean ± SD) in average peak torque over the sets during the slow-eccentric exercise (ECC-S and ISO + ECC-S, two sets of maximal slow-eccentric contractions) and fast-eccentric exercise (ECC-F and ISO + ECC-F, six sets of maximal fast-eccentric contractions). (*) *p* < 0.05 for ECC-F vs. ISO + ECC-S, (a) *p* < 0.05 from the first set for ECC-S and ISO + ECC-S, (b) *p* < 0.05 from the first set for ECC-F and ISO + ECC-F, (c) *p* < 0.05 from the previous set for ECC-F, and (d) *p* < 0.05 from the previous set for ISO + ECC-F.

**Figure 3:**
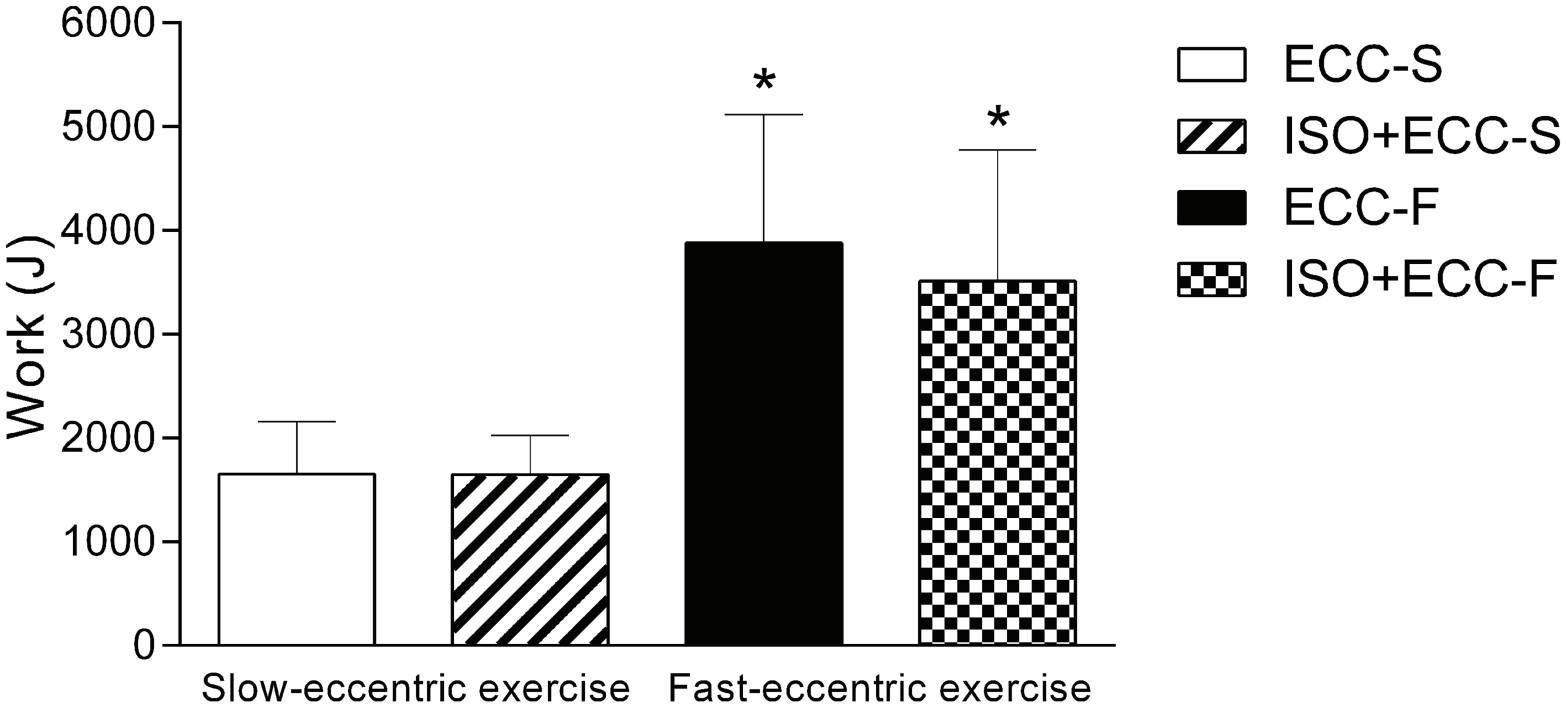
Average total work (mean ± SD) of slow- (ECC-S and ISO + ECC-S) and fast-eccentric (ECC-F and ISO + ECC-F) exercises. (*) *p* < 0.05 vs. ECC-S and ISO + ECC-S.

Average peak torque of the elbow flexors during the eccentric exercise protocols (slow and fast eccentric exercise) was similar between groups (ECC-S: 47.5 ± 12.9 N·m, ECC-F: 49.6 ± 10.2 N·m, ISO + ECC-S: 46.9 ± 9.6 N·m, ISO + ECC-F: 46.3 ± 13.5 N·m) (*p* = 1). Figure 2 shows average peak torque over the sets of slow and fast eccentric exercise. There was a significant difference (*p* < 0.05) between ECC-F and both slow eccentric exercise groups (ECC-S and ISO + ECC-S) on the second set of maximal eccentric exercise. Average peak torque decreased over the sets for all groups (*p* < 0.05). Total work was similar within slow eccentric exercise groups (ECC-S: 1617.2 ± 509.7 J, ISO + ECC-S: 1648.3 ± 379.6 J) and fast eccentric exercise groups (ECC-F: 3875.3 ± 1199.2 J, ISO + ECC-F: 3510.9 ± 1267.9 J), but different between both velocity conditions (*p* < 0.05) (Figure 3).

### Changes in MVC

**Figure 4:**
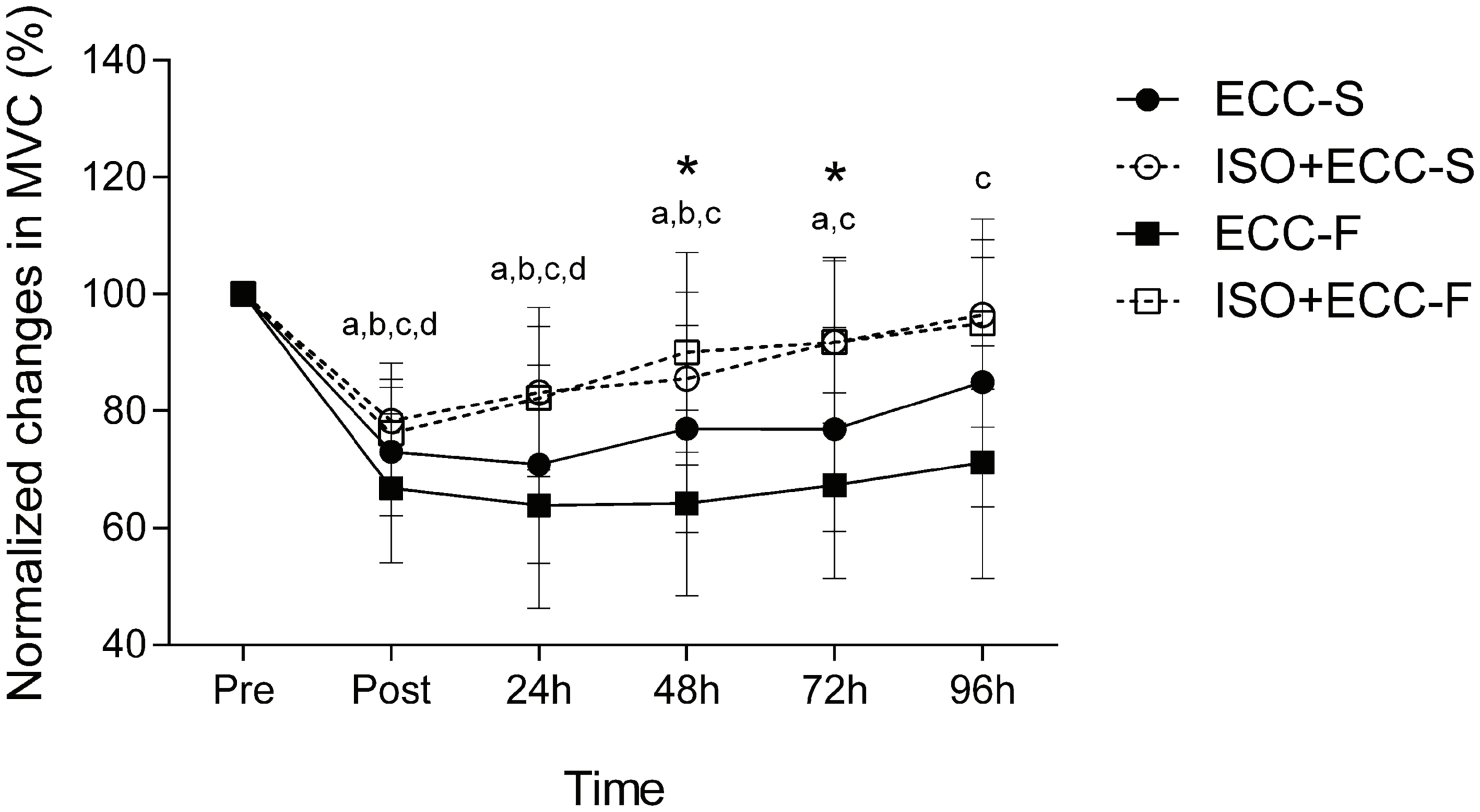
Normalized changes (mean ± SD) in maximal voluntary concentric contraction peak torque before (Pre), immediately after (Post) and 24–96 h after maximal eccentric exercise. (*) *p* < 0.05 for ECC-F vs. ISO + ECC-F, (a) *p* < 0.05 vs. Pre for ECC-S, (b) *p* < 0.05 vs. Pre for ISO + ECC-S, (c) *p* < 0.05 vs. Pre for ECC-F, and (d) *p* < 0.05 vs. Pre for ISO + ECC-F.

Significant (*p* < 0.05) group vs. time interaction was found for MVC (*F* = 4,517). Figure 4 shows percentage changes in MVC immediately after, and for 4 days following MaxECC for all groups. MVC was significantly compromised (*p* < 0.05) for all groups immediately after eccentric exercise (ECC-S: −27 ± 11%, ISO + ECC-S: −22 ± 10%, ECC-F: −33 ± 13%, ISO + ECC-F: −24 ± 9%). Recovery was significantly faster for ISO + ECC-S and ISO + ECC-F compared with the control groups, and recovery was faster for ISO + ECC-F than ISO + ECC-S. Significant differences were found in MVC between ECC-F and ISO + ECC-F at 48 and 72 h post-MaxECC (*p* < 0.05). No significant differences between control groups were evident (*p* = 1).

### Changes in SOR

**Figure 5:**
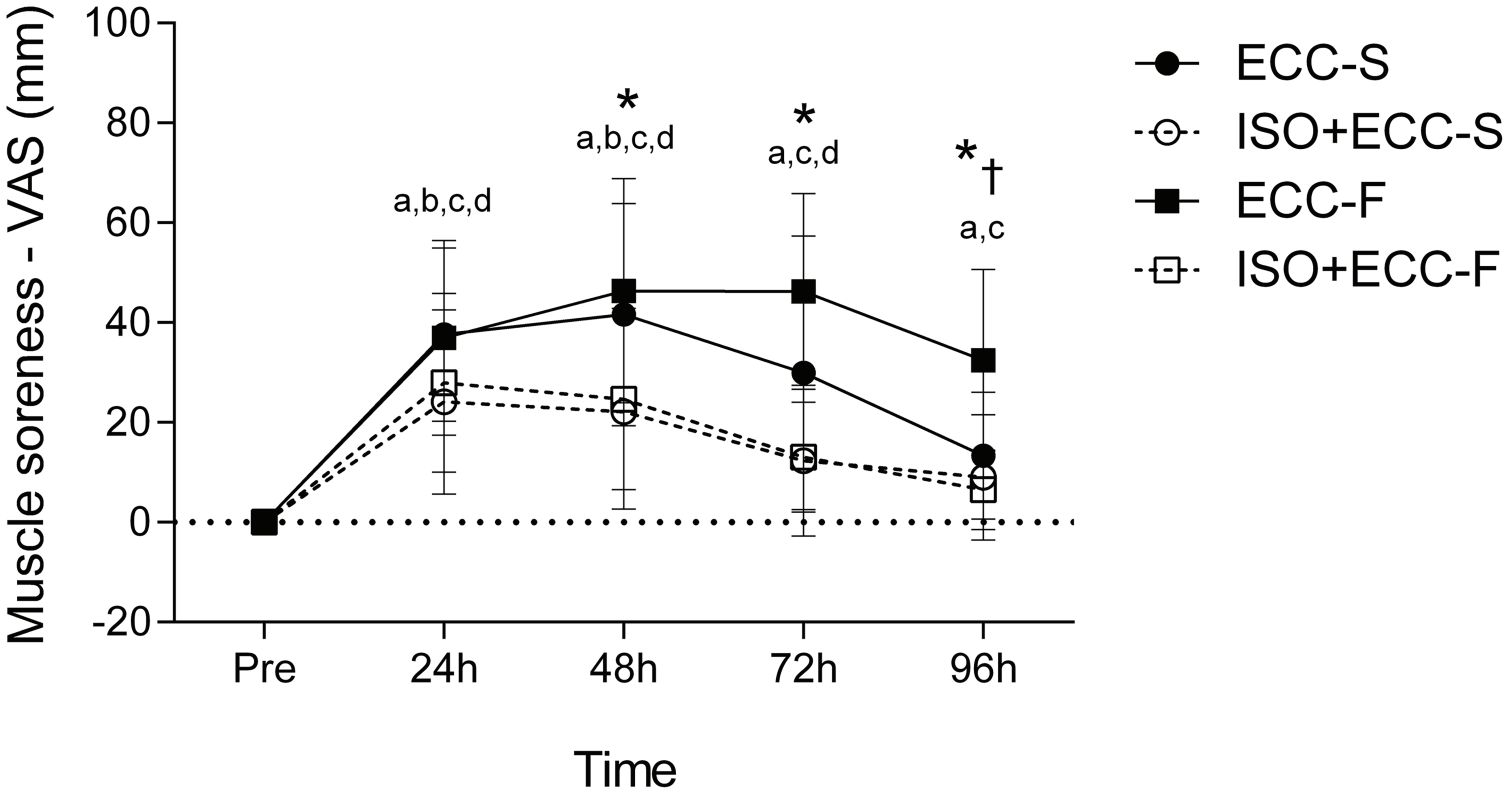
Absolute changes (mean ± SD) in SOR before (Pre) and 24–96 h after maximal eccentric exercise. (*) *p* < 0.05 for ECC-F vs. ISO + ECC-F, (†) *p* < 0.05 for ECC-S vs. ECC-F, (a) *p* < 0.05 vs. Pre for ECC-S, (b) *p* < 0.05 vs. Pre for ISO + ECC-S, (c) *p* < 0.05 vs. Pre for ECC-F, and (d) *p* < 0.05 vs. Pre for ISO + ECC-F.

Significant (*p* < 0.05) group vs. time interaction was found for SOR (*F* = 6,318). SOR increased significantly (*p* < 0.05) on the days following MaxECC for all groups. SOR was greater (*p* < 0.05) for ECC-F than ECC-S at 96 h post-MaxECC. The magnitude of the development of SOR after MaxECC was significantly smaller for ISO + ECC-F compared with its control (ECC-F) at 48–96 h (Figure 5). Recovery was significantly faster for ISO + ECC-S and ISO + ECC-F compared to the control groups, and recovery of ISO + ECC-S was faster than the ISO + ECC-F. No significant differences between ECC-S and ISO + ECC-S were identified (*p* = 0.66).

### Changes in MT

**Figure 6:**
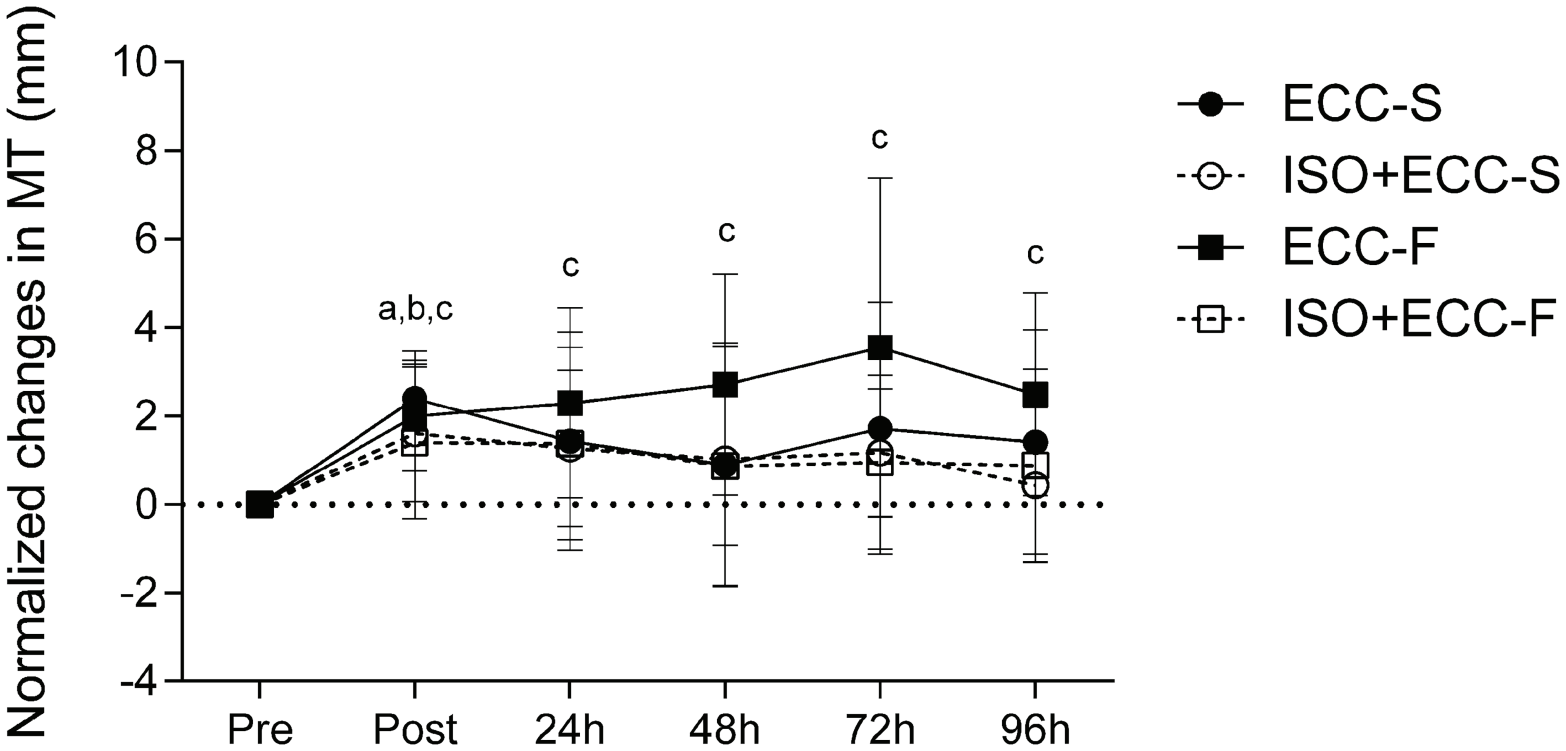
Normalized changes (mean ± SD) in MT before (Pre), immediately after (Post), and 24–96 h after maximal eccentric exercise. (a) *p* < 0.05 vs. Pre for ECC-S, (b) *p* < 0.05 vs. Pre for ISO + ECC-S, (c) *p* < 0.05 vs. Pre for ECC-F, and (d) *p* < 0.05 vs. Pre for ISO + ECC-F.

Significant (*p* < 0.05) group vs. time interaction was found for MT (*F* = 1,863). MT increased significantly (*p* < 0.05) immediately after eccentric exercise for both control groups and ISO + ECC-S. ECC-F presented increased MT (*p* < 0.05) until 96 h post-MaxECC, whilst ECC-S and ISO + ECC-S reached full recovery at 24 h after exercise. MT was not significantly affected by the damaging protocol for ISO + ECC-F (Figure 6). No significant differences were found between groups for this dependent variable (*p* = 1).

### Index of Protection

**Figure 7:**
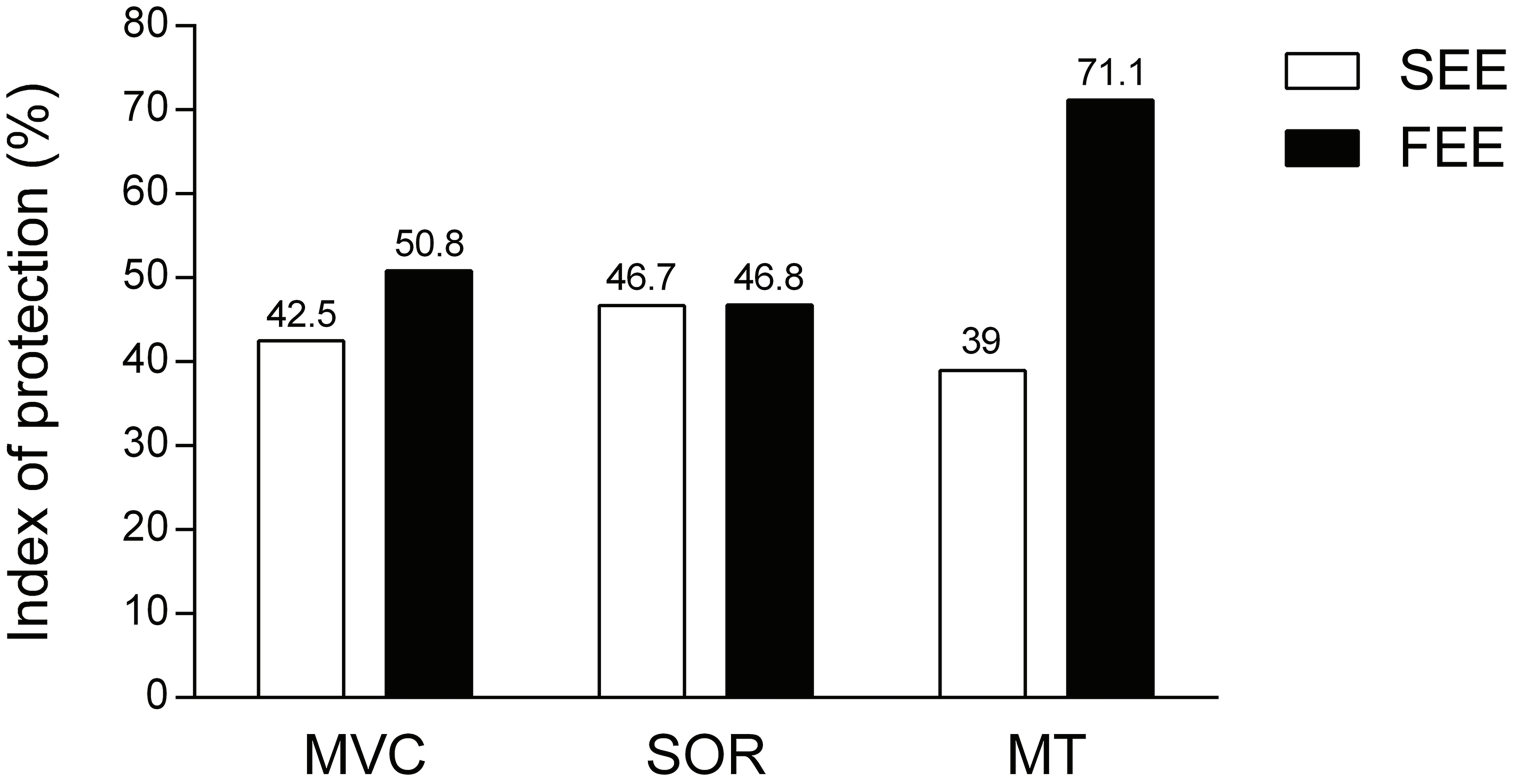
Index of protection of isometric preconditioning on the two conditions of muscle damage. Indexes of protection are expressed as percentage (%) for groups that performed slow-eccentric exercise (SEE) or fast-eccentric exercise (FEE) for maximal voluntary concentric contraction (MVC), muscle soreness (SOR), and muscle thickness (MT) variables, based on the following equation: (percentage change in the variable for the control group − percentage change in the variable for the experimental group)/percentage change in the variable for the control group × 100. The greater the percentage value, the greater the protective effect induced by isometric preconditioning.

The protective effect was apparently greatest against EIMD induced by fast-velocity eccentric exercise, as evidenced by higher IP for MVC and MT. The protective effect seems similar between the different conditions of EIMD (induced by slow- or fast-velocity eccentric exercise) for SOR (Figure 7).

## Discussion

The main purpose of this study was to investigate whether an IPP would similarly attenuate the magnitude of change in EIMD markers induced by different eccentric exercise protocols. The hypotheses tested were as follows: 1) the IPP would attenuate EIMD by slow and fast eccentric exercise and 2) the protection conferred by the IPP would be lesser against EIMD by fast eccentric exercise. The obtained results confirmed the hypothesis that IPP would attenuate EIMD induced by slow and fast eccentric exercise but rejected the hypothesis that protection would be greater against EIMD by slow eccentric exercise.

The results showed that there was a manifestation of EIMD in the elbow flexors of the participants in all groups, evidenced by a decrease in MVC, development of SOR, and increase in MT of the exercised limb after both eccentric exercise protocols, in accordance with previous studies (Nosaka and Sakamoto, 2001; Chen et al., 2011, 2012b). Changes in dependent variables following damaging bout for the control groups indicate that the two eccentric exercise protocols investigated induced different extents of EIMD. Although no significant difference was found between control groups for MVC and MT, participants who performed fast eccentric exercise showed greater SOR at 96 h post-MaxECC and a delayed recovery of all assessed variables as compared to those who performed slow eccentric exercise (Figures 4–6). Thus, based on the different recovery kinetics observed between ECC-F and ECC-S, it is evident that the two different eccentric exercise protocols induced different changes on EIMD markers, supporting what has been previously presented in the literature (Chapman et al., 2006, 2008) and corroborating one of our hypotheses.

The extent of EIMD can be assessed by either the magnitude of changes in direct and indirect markers of EIMD, but also the time-course of recovery of these markers. Several studies found different extents of EIMD evidenced by different recovery kinetics of indirect markers of EIMD (Hirose et al., 2004; Nosaka et al., 2005, 2006; Paddon-Jones et al., 2005). For instance, studies that investigate the RBE often find accelerated recovery of muscle function without differences in the magnitude of changes in it following damaging bouts. Paddon-Jones et al. (2005) showed that different eccentric contraction velocities (30 and 180°·s^−1^) induced different degrees of EIMD. In their study, slow and fast eccentric exercise protocol had a similar effect on plasma CK activity and isometric torque; however, the differences in kinetics recovery of concentric and eccentric torque, muscle swelling, and SOR suggested that there were different extents of EIMD (Paddon-Jones et al., 2005).

In light of our results, it seems that the difference in MaxECC velocities was not a strong factor that affected EIMD severity. It is important to note that Chapman et al. (2006) also found smaller changes in indirect markers of EIMD after slow-velocity MaxECC than fast-velocity MaxECC when total time under tension was equated. As well as in the study by Chapman et al. (2006), the same time under tension was used as the criterion to match protocols of slow- and fast-velocity eccentric exercise in the present study. Nonetheless, the time under tension chosen by Chapman et al. (2006) (120 s) was greater than that used in the present study (45 s). Consequently, the number of contractions used in the study by Chapman et al. (2006) was greater (30 MaxECC for slow angular velocity and 210 MaxECC for fast angular velocity) compared to the volume used in the eccentric exercise protocols of this study (30 MaxECC for slow eccentric exercise and 90 MaxECC for fast eccentric exercise). The obtained results suggest that the effect of contraction velocity on EIMD magnitude is more pronounced when MaxECC protocols have a substantial time under tension (more than 45 s). Furthermore, Chapman et al. (2006, 2008) used eccentric exercise protocols with more extreme angular velocities, such as 30 and 210°·s^−1^ (i.e., angular velocity of fast exercise seven times greater than slow exercise), to induce different magnitudes of EIMD, whilst the velocities chosen for the MaxECC protocols of the present study were closer; 60 and 180°·s^−1^ (i.e., angular velocity of fast exercise three times greater than slow exercise). In our study, fast-eccentric exercise had an angular velocity and number of contraction three times greater than the slow eccentric exercise protocol, so, although not large, the observed differences in the time-course of recovery of markers of EIMD seen after slow- and fast-velocity MaxECC are attributable to both contraction velocity and number of contractions, which is in line with findings of Chapman et al. (2008).

Regarding the efficacy of IPP in blunting changes in markers of EIMD, participants in the ISO + ECC-S and ISO + ECC-F groups – which performed this strategy – presented a smaller decrease in MVC, increase in SOR, and faster recovery of all variables compared to their respective control groups (ECC-S and ECC-F), demonstrating the effectiveness of IPP in protecting against subsequent EIMD. These findings corroborate previous studies which demonstrated the efficacy of IPP as a strategy for attenuation of EIMD by eccentric exercise in the elbow flexors (Chen et al., 2012a,b, 2013).

In fact, Chen et al. (2012a) found smaller protection against strength loss (IP = 26.9%) and greater protection against SOR (IP = 74.9%) using an identical IPP (10 MaxISO at 20° of elbow flexion) against EIMD induced by 30 MaxECC at 90°·s^−1^, than the protection found for both conditions of EIMD severity in the present study (MVC: slow eccentric exercise IP = 42.5% and fast eccentric exercise IP = 50.8%; SOR: slow eccentric exercise IP = 46.7%; and fast eccentric exercise IP = 46.8%). The distinction in magnitudes of protection between these studies may be due to inter-individual variability in EIMD outcomes, such as training status (Newton et al., 2008) and/or genetic factors (Clarkson et al., 2005). Moreover, Chen et al. (2012a) induced muscle damage with a different eccentric exercise protocol (30 MaxECC at 90°·s^−1^), and as already stated, it has not yet been established if the protective effect conferred by IPP is the same against different damaging protocols.

Concerning the magnitude of the protective effect conferred by the same IPP against different extents of EIMD (induced by slow- or fast-velocity eccentric exercise), the groups which performed the fast-velocity eccentric exercise protocol benefited from a greater protective effect in all variables, indicating that IPP was more effective in the condition of greater EIMD. As mentioned above, the IP value for MVC was greater against muscle damage induced by fast- than slow-velocity eccentric exercise (50.8 vs. 42.5%, respectively). Moreover, strength loss was greater (*p* < 0.05) for ECC-F compared to ISO + ECC-F at 48 and 72 h following the damaging bout, whilst there was no significant difference between ECC-S and ISO + ECC-S at any time point. The mechanisms proposed to explain the protective effect conferred by the IPP in muscle function are related to mechanical adaptations of the muscle-tendon complex, such as increased muscle compliance (Kay and Blazevich, 2009), or nervous system-related adaptations, such as an increased neural drive and improvements in the synchronization of motor units of the agonist and antagonist muscles during the subsequent MaxECC (Green et al., 2014). It has been proposed that ATP availability decreases during repetitive eccentric contractions leaving the skeletal muscle in a state of rigor, increasing strain imposed on active muscle fibers, and resulting in disruption of sarcomere structures (Morgan, 1990). A greater amount of fibers may enter a state of rigor when greater numbers of contractions are performed. Thus, it is possible that improved motor unit recruitment induced by IPP may collaborate to reduce fatigue, which could provide greater protection against the eccentric exercises protocol with a large number of contractions. However, Figure 2 shows that average peak torque and the extent of torque decrement were similar between control groups and their respective experimental groups over sets of maximal eccentric exercise protocols. Therefore, it is unlikely that adaptations induced by the IPP promote great protection against the primary, tensional, damage, and such adaptations do not seem to explain the greater protective effect observed in the severe EIMD condition.

The protective effect against muscle swelling conferred by IPP was greater in the fast-eccentric exercise condition than slow-eccentric exercise condition, as evidenced by a greater IP value of the former (71.1 vs. 39.0%, respectively). Furthermore, this symptom was not significantly affected by the damaging bout for the ISO + ECC-F group, whilst its respective control group (ECC-F) presented increased MT until the last day of the experiment. On the other hand, the ISO + ECC-S group reached full recovery at the same moment as its respective control group (ECC-S). The IP values related to SOR were apparently similar between the groups that performed slow and fast eccentric exercise (46.7 and 46.8%, respectively). However, a significant difference between the control and IPP groups was only found for the groups which performed fast eccentric exercise (i.e., which suffered from greater extent of EIMD). Such findings suggest, again, a greater protective effect conferred by IPP against more severe EIMD.

The mechanisms responsible for the protective effect conferred by IPP, especially on muscle swelling and SOR, are mostly related to adaptations in the inflammatory process and attenuation of secondary damage (Lima and Denadai, 2015). Changes in gene expression related to reactive oxygen species, increased expression of heat shock proteins (HSP), and greater accumulation of neutrophils in the musculature following maximal isometric exercise are findings that suggest a more robust protective effect in the second phase of EIMD (Pizza et al., 2002; McArdle et al., 2004; Nagahisa et al., 2018).

Pizza et al. (2002) showed that maximal isometric contractions lead to accumulation of neutrophils in muscle tissue without the manifestation of tissue damage in an animal model. McArdle et al. (2001) found increased expression of HSP (i.e., HSP60 and HSP7) when performing 164 isometric contractions on rat soleus muscle. Interestingly, increased expression of such proteins started between 2 and 4 h following the isometric exercise – with a peak between 18 and 48 h – and remained until 72 h later. This window coincides with the IPP window, which induces short-term adaptations (1 or 2 days), is short-lived (lasting approximately 4 days), and induces a potent protective effect against EIMD a few days later (Chen et al., 2013). McArdle et al. (2004) found increases in haemoxygenase-1 gene expression and increased production of reactive oxygen species induced by an IPP which may also play an important role in the mechanisms of protection against EIMD – specifically on secondary damage. Moreover, in a recent study, Nagahisa et al. (2018) found smaller increases in mRNA expression of molecules related to SOR (i.e., BKB2 receptor, COX-2, and mPGEC-1), when a small volume of eccentric contractions (10 contractions) was performed 2 days before a damaging protocol consisting of 100 eccentric contraction in the rat plantar flexor muscles when compared to a control group that did not undergo any preconditioning.

In our study, the main differences between the two conditions of EIMD severity were observed in the recovery phase, which is related to the magnitude of secondary, inflammatory damage. Moreover, a greater increase in arm circumference and SOR after fast eccentric bout compared to slow eccentric bout (with same number of contractions – 210 MaxECC), showed by Chapman et al. (2008), suggests that contraction velocity may have a greater influence on the magnitude of the inflammatory phase of EIMD. Therefore, an acute inflammatory response directed to the stressed tissues and changes in gene expression related to indirect markers of EIMD induced by MaxISOs may have contributed to the attenuation/faster time-course of recovery of markers of EIMD following the fast-eccentric exercise protocol (Deyhle et al., 2016).

It is important to emphasize that the theoretical construct regarding the mechanisms involved in IPP was mostly developed in animal models, whilst studies investigating functional effects of this phenomenon were conducted in humans. This reveals a dichotomy between the mechanistic and applied understanding of isometric preconditioning, as well as a lack of randomized clinical trials focusing on the physiological mechanisms that would explain IPP in humans. We did not assess neural aspects of muscle contractions, muscle-tendon complex behavior, or inflammatory markers that could elucidate the mechanistic bases of the greater protective effect conferred by IPP against faster MaxECC. Instead, the present study demonstrates that the short-term adaptations induced by MaxISO can be more potent in protecting against a larger number of eccentric contractions at higher angular velocities.

Thus, when performed before fast eccentric exercise (90 MaxECC at 180°·s^−1^), the IPP attenuated the magnitudes of strength loss and SOR and led to faster recovery for all variables (MVC, SOR, and MT) whilst not inducing any protective effect regarding muscle swelling and only accelerating recovery (without attenuating the magnitude of changes) of strength loss and SOR when performed prior to slow eccentric exercise (30 MaxECC at 60°·s^−1^). Whilst rejecting one of our hypotheses – that IPP would promote a greater magnitude of protection against the exercise that represents less stress to the musculature – these results provide the first evidence that the magnitude of the protective effect conferred by IPP is dependent on the subsequent exercise effort.

From a practical perspective, isometric preconditioning can be an interesting strategy for coaches aiming to improve the adherence of novice individuals that undertake different resistance-training regimens – where some EIMD markers such as SOR and swelling are undesired. Further studies are necessary to investigate the scope of such a protective effect in practical terms. For instance, it would be interesting to investigate whether IPP would promote a potent protection in the sports competition field, which involves faster eccentric actions than in traditional resistance training conditions in populations that are already resistant to EIMD.

As limitations, it can be pointed out that no serum marker of inflammation or extravasation of intramuscular proteins was evaluated. Another possible limitation of the study may be related to the experimental design adopted, which did not include pre- and post-IPP assessments to observe if the IPP induced any change in EIMD markers before eccentric exercise. However, the literature (Chen et al., 2012a, 2013) has already shown that the very same IPP does not induce significant increases in markers of EIMD in the same muscle group. Nonetheless, this is the first study to demonstrate that the magnitude of the protective effect conferred by an IPP is also dependent on the subsequent exercise, with a greater protective effect conferred against maximal fast-velocity eccentric contractions.

In conclusion, the present study showed that IPP confers a potent protective effect against different extents of EIMD in untrained young men. The efficacy of IPP seems to be greater in the attenuation of the severest EIMD conditions. Furthermore, IPP can be used as an interesting strategy to attenuate and accelerate the recovery of muscle damage induced by different eccentric exercise protocols.

## Data Availability Statement

The datasets generated for this study are available on request to the corresponding author.

## Ethics Statement

This study was reviewed and approved by Ethics Committee on Human Research of the Institute of Biosciences of São Paulo State University - Rio Claro. The patients/participants provided their written informed consent to participate in this study.

## Author Contributions

RB, LL, CG, and BD contributed the conception and design of the study. RB organized the database. RB, LL, and BD performed the statistical analysis. RB wrote the first draft of the manuscript. RB and LL wrote sections of the manuscript. All authors contributed to manuscript revision, read, and approved the submitted version.

### Conflict of Interest

The authors declare that the research was conducted in the absence of any commercial or financial relationships that could be construed as a potential conflict of interest.
